# Management of malignant pleural mesothelioma – part 1: epidemiology, diagnosis, and staging: Consensus of the Austrian Mesothelioma Interest Group (AMIG)

**DOI:** 10.1007/s00508-016-1080-z

**Published:** 2016-09-12

**Authors:** Christian Geltner, Peter Errhalt, Bernhard Baumgartner, Gerhard Ambrosch, Barbara Machan, Josef Eckmayr, Thomas Klikovits, Mir Alireza Hoda, Helmut Popper, Walter Klepetko, Austrian Mesothelioma Interest Group (AMIG)

**Affiliations:** 1 Department of Pulmonology, Klinikum Klagenfurt, Feschnigstraße 11, Klagenfurt, Austria; 2 Department of Pulmonology, University Clinic Krems, Krems, Austria; 3 Department of Pulmonology, Landeskrankenhaus Vöklabruck, Vöklabruck, Austria; 4 Department of Pulmonology, Landeskrankenhaus Graz Südwest, Graz, Austria; 5 Rehabilitation Center Tobelbad, Allgemeine Unfallversicherungsanstalt, Tobelbad, Austria; 6 Department of Pulmonology, Landeskrankenhaus Wels, Wels, Austria; 7 Division of Thoracic Surgery, Department of Surgery, Comprehensive Cancer Center, Medical University Vienna, Währinger Gürtel 18–20, 1090 Vienna, Austria; 8 Department of Pathology, Medical University Graz, Graz, Austria; 9 Division of Thoracic Surgery, Department of Surgery, Comprehensive Cancer Center, Medical University Vienna, Waehringer Guertel 18–20, 1090 Vienna, Austria

**Keywords:** Malignant pleural mesothelioma, Epidemiology, Staging, Pathology, Diagnosis

## Abstract

Malignant pleural mesothelioma is a rare malignant disease that in the majority of cases is associated with asbestos exposure. The incidence in Europe is about 20 per million inhabitants and it is increasing worldwide. Initial symptoms are shortness of breath, pleural effusion, cough, and chest pain. The typical growth pattern is along the pleural surface; however, infiltration of the lung and/or mediastinal and chest wall structures can occur in a more advanced stage. Ultimately, distant metastases outside the chest can result. Several histological subtypes of pleural mesothelioma exist, which must be differentiated from either benign diseases or metastases in the pleural space by other tumor entities. This differential diagnosis can be very difficult and a large panel of immunohistochemical markers is required to establish the exact diagnosis. The standard procedure for confirming the disease and obtaining sufficient tissue for the diagnosis is videothoracoscopy. Full thickness biopsies are required, while transthoracic needle puncture of pleural fluid or tissue is considered to be insufficient for a cytological diagnosis. Complete and detailed staging is mandatory for categorization of the disease as well as for therapeutic decision making.

## Epidemiology

Asbestos exposure is the main risk factor for the development of malignant pleural mesothelioma (MPM) [1]. Asbestos comprises silicate minerals with very thin fibers: chrysotile, crocidolite as serpentines, amosite, anthophyllite, tremolite, and actinolite from the amphibole group [2]. Chrysotile is biologically active and detectable in the lungs for a shorter time. Chrysotile, amosite, and crocidolite were mined and used in ship and railway construction as well as in fire protection engineering. The first evidence of their high carcinogenic potential was found in the UK and South Africa as early as the 1960s [3]. Amosite and crocidolite seem to have a higher carcinogenicity than the other types of asbestos [2]. Asbestos exposure is typically labor-dependent and is recognized as an occupational disease. More recently, a shift has been observed from asbestos-removal workers to professionals involved in post-construction work, e. g., electricians, plumbers, or heat protection technicians. This is paralleled by a profession-dependent gender distribution, as more than 80 % of affected individuals are men [4].

“Environmental” occurrence of mesothelioma has been found in people growing up in the vicinity of natural asbestos resources (Turkey, Corsica, Cyprus) or in areas where asbestos was used for the whitening of house walls. The load in rooms built with asbestos-containing materials was initially seen as hazardous. The actual resulting asbestos dose is, however, extremely low and carcinogenic levels are not detectable [5].

Furthermore, there is an increasing incidence of nonoccupational asbestos disease among housewives and family members of asbestos workers as well as a high environmental impact in the vicinity of mining and processing facilities [6, 7].

There is a clear correlation between the amount of asbestos exposure and the incidence of MPM. The mean latency period between exposure to asbestos and the onset of symptoms is up to 40 years, and 99 % of cases show a latency of more than 15 years [8].

The occurrence of MPM is independent of other asbestos-associated diseases such as classic asbestosis of the lung (interstitial lung disease with fibrosis) and benign pleural plaques. Although pleural plaques are also associated with asbestos exposure, studies from Australia could not find any connection between plaques and an increased incidence of MPM [9].

The incidence of MPM varies between 7 per million inhabitants in Japan and 40 per million in Australia. In Europe, the average incidence is 20 per million inhabitants. The frequency is highly dependent on the amount of asbestos removal, asbestos import, and industrialization. In Europe, peak incidence is to be expected between 2015 and 2020 due to the long latency period [10].

By contrast, the incidence of mesothelioma without asbestos contact is extremely low (<1: 1 million). Other potential cofactors for the development of mesothelioma besides asbestos are synthetic materials (ceramics, nanoparticles), ionizing radiation, and SV-40 virus infections [11].

The impact of cigarette smoke as well as numerous other fibrous materials such as glass fibers and mineral glass wool is, however, excluded. Genetic factors may play a role, since both familial clustering and endemic accumulation in populations with high natural exposure are known in Turkey [5, 12].

**Fig. 1:**
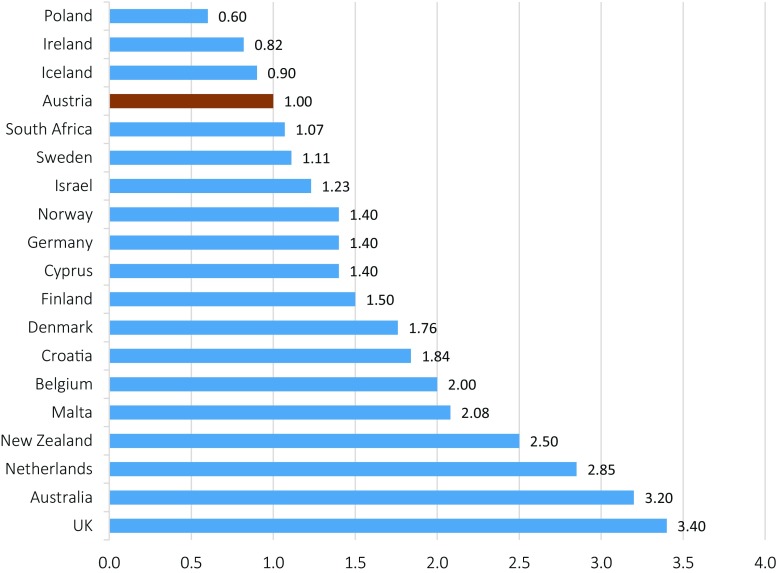
Incidence of MPM in comparison to Austria [25]

In Austria, there have been 276 cases of MPM approved by the AUVA (*Allgemeine Unfallversicherungsanstalt* – General accident insurance company) as being caused occupationally within the last 5 years. Of these, 53 were approved in 2014 only. In contrast to this, ten asbestos-related MPM cases were documented in 1995 and 41 in 2005. However, there is still uncertainty about the number of MPM cases not being reported to the AUVA. A comparison of the incidence of MPM worldwide and in Austria is depicted in Fig. 1.

## Screening

In the current European Society of Thoracic Surgery (ESTS)/European Respiratory Society (ERS) guidelines no general screening methods are recommended [13]. This is based on the low sensitivity of even advanced imaging techniques such as low-dose computed tomography (CT) in screening of asbestos workers.

Circulating biomarkers, such as osteopontin, mesothelin-related peptides, and soluble mesothelin-related peptide (SMRP) [14], and fibulin-3 [15] have also been evaluated in MPM extensively. However, none of them are considered to be a reliable screening tool, since false-positive results are too frequent [16].

## Diagnosis

### Clinical symptoms

Specific symptoms of MPM are dyspnea, cough, and chest pain on initial examination. Shortness of breath is often initially caused by a pleural effusion and later by extensive restriction due to pleural and pulmonary tumor masses in the thoracic cavity. Patients describe chest pain as diffuse, sometimes radiating into the shoulders, arms, or abdomen. Tumor ingrowth into the neural structures of the brachial plexus and the intercostal or paravertebral structures can also cause neuropathic pain. Weight loss is a symptom of more advanced disease.

Typically, MPM occurs initially unilaterally. The tumor can, however, spread to the other pleural cavity or into the peritoneum in the further course of disease. Compared with lung cancer, distant metastases in the extrathoracic lymph nodes or in other parenchymal organs are usually rare, although they do occur in very advanced stages [17].

### Diagnostic procedures

**Fig. 2:**
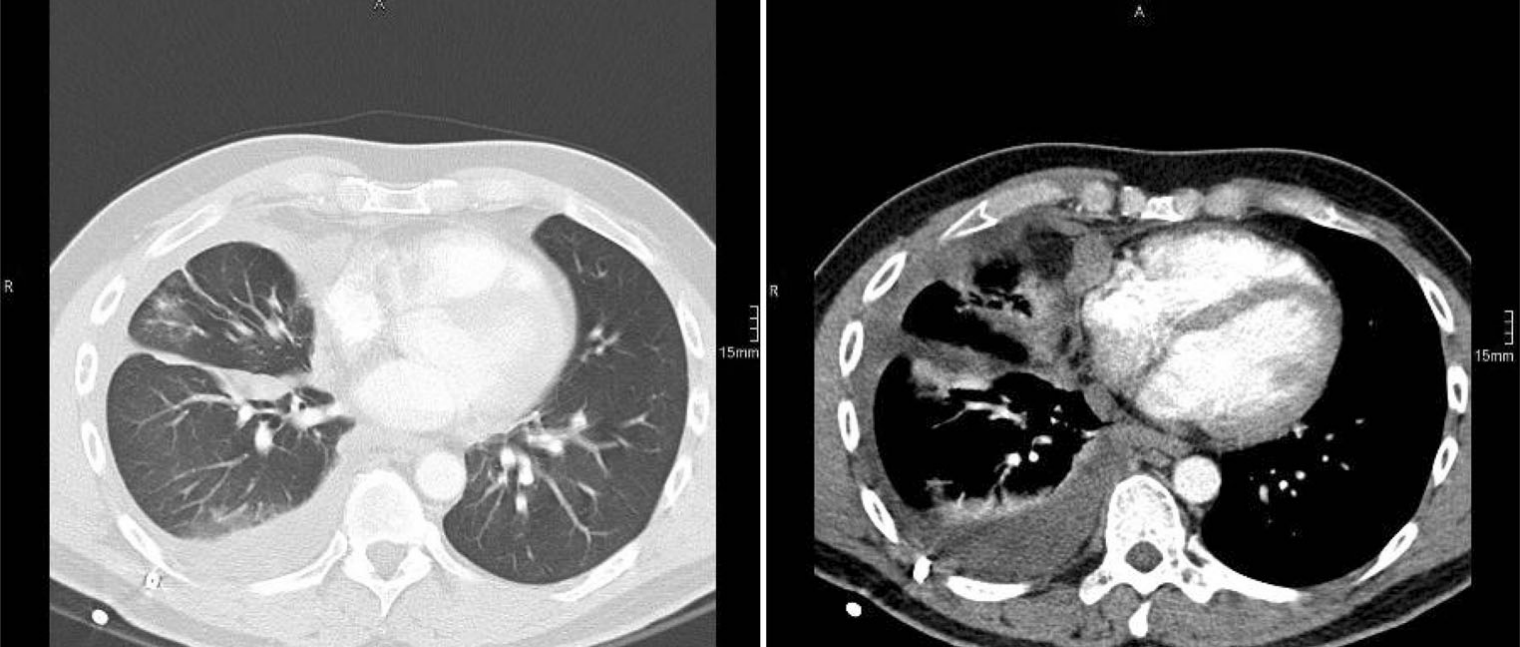
Computer tomography of patient with malignant pleural mesothelioma showing circular involvement of the voscera and parietal pleura, pericardium and mediastinum. Pulmonary window (*left*) and mediastinal window (*right*)

The typical finding on chest X‑rays of patients with MPM is pleural effusion or pleural thickening, which, however, is not specific. CT scan (Fig. 2) is more helpful, but it still does not allow a definitive diagnosis to be made since its sensitivity reaches only 40 %, and in most cases it does not distinguish between benign and malignant processes. The same holds true for positron emission tomography (PET)-CT where no clear standardized uptake value (SUV) level is considered suggestive for MPM and false-positive results can occur in other processes such as tuberculosis, parapneumonic effusions, and uremia [18].

Puncture of the pleural effusion that is usually present and cytological examination of the fluid is also not conclusive, especially since the differential diagnosis with pleural metastases of other tumors such as bronchial or breast cancer (e. g., adenocarcinomas) cannot be made in most cases. Also the macroscopic aspect of MPM is so variable that simple thoracoscopy does not confirm the diagnosis.

**Fig. 3:**
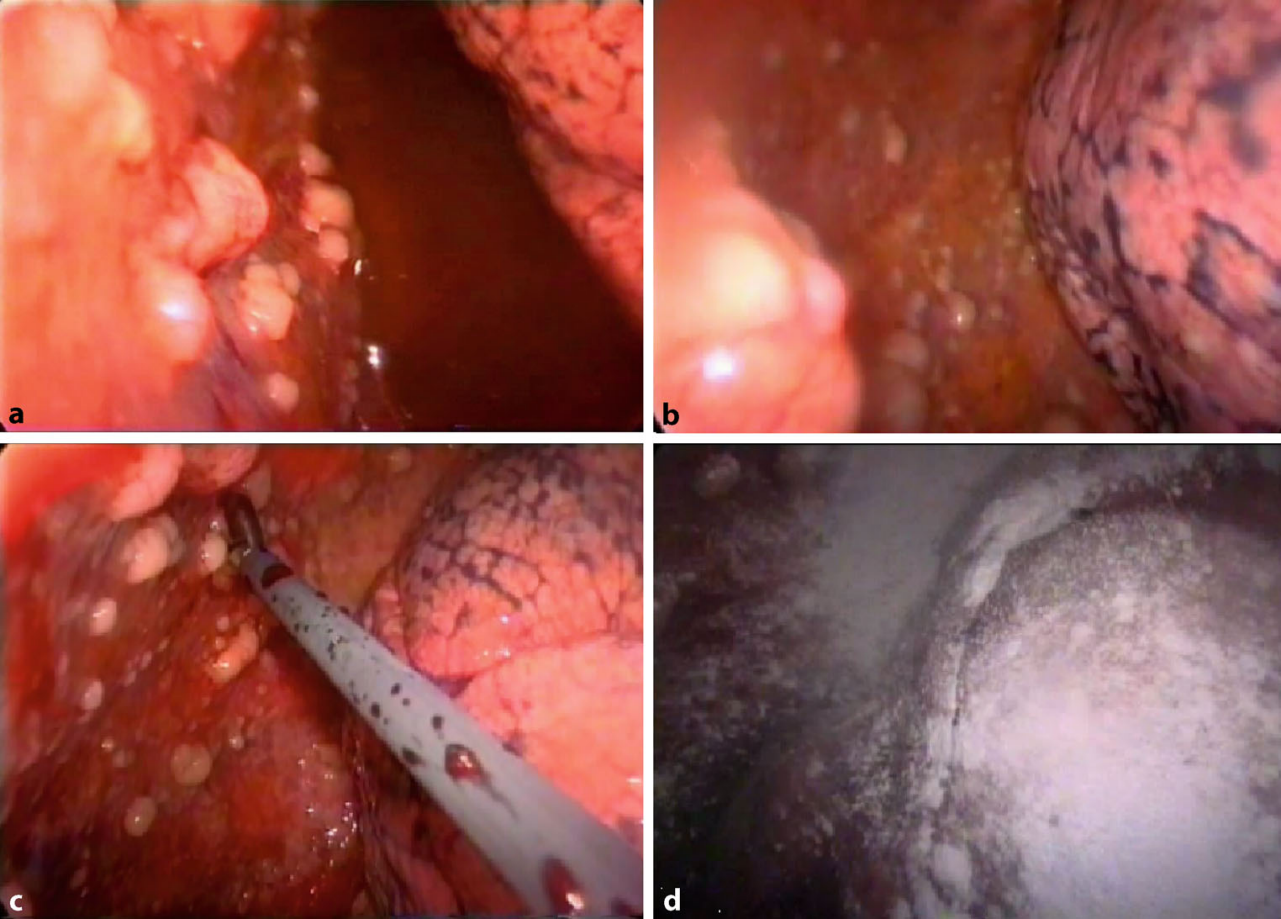
Video-assisted thoracoscopic view of MPM mainly on the parietal pleura (**a,** **b**), forceps biopsy (**c**), and talc pleurodesis (**d**)

For these reasons, the precise diagnosis of MPM requires a histopathological confirmation and thoracoscopy remains the standard procedure for obtaining tissue and performing macroscopic staging of the pleural tumor spread at the same time (Fig. 3). Thoracoscopy can be performed with the patient under local anesthesia or as video thoracoscopy via a surgical approach. This allows one to combine the diagnostic procedure with the initial therapeutic step of talc pleurodesis (Fig. 3). Only in exceptional situations should a CT- or ultrasound-guided fine needle biopsy be performed, which, however, has a a clearly lower sensitivity.

**Fig. 4:**
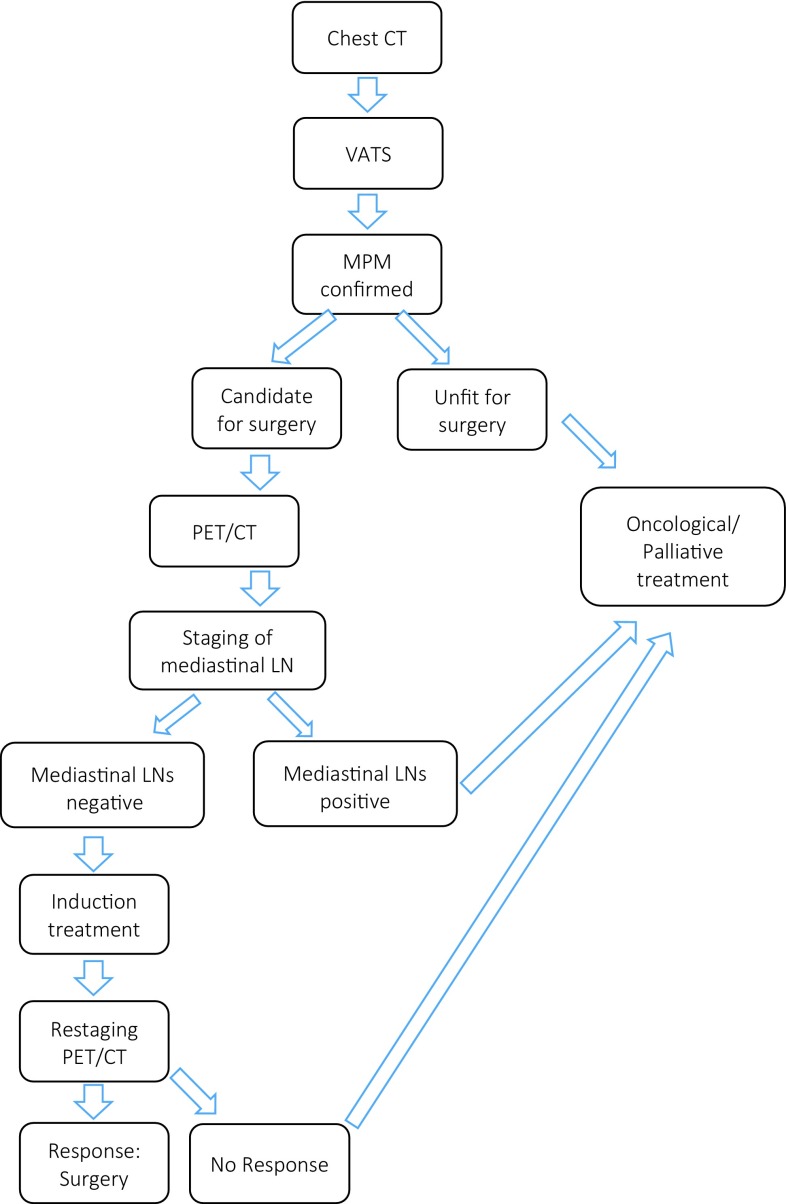
Proposed staging algorithm for MPM patients in Austria

The typical diagnostic algorithm applied in most centers around the world is displayed in Fig. 4.

### Histopathology

**Fig. 5:**
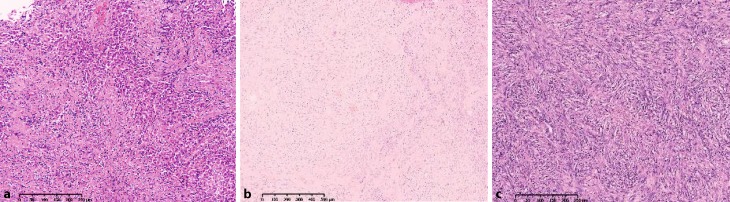
Examples of malignant pleural mesothelioma (MPM): epithelioid MPM **a**), biphasic MPM (**b**), and sarcomatoid MPM (**c**). (Kindly provided by Dr. Luka Brcic, Department of Pathology, Medical University Graz)

MPM derives from the pleural stem cell, which exhibits epithelioid and sarcomatoid growing patterns at the same time. Depending on which component is predominant, three histological types of MPM can be distinguished: epithelioid (50–70 %), sarcomatoid (7–20 %), and a mixed or biphasic form (20–35 %; Table 1 and Fig. 5).

**Table 1:** Histological specification of malignant pleural mesothelioma [23]

Epithelioid	Sarcomatoid	Biphasic mixed
– Tubulopapillary – Acinar – Glandular – Adenomatoid – Solid epithelioid patterns – Small cell – Oat cell Differential diagnosis: metastatic carcinomas and other epithelioid tumors	Mimic malignant mesenchymal tumors: leiomyosarcoma synovial sarcoma Desmoplastic mesothelioma bland tumor cells Differential diagnosis: sarcomatoid carcinoma and other sarcomas	Combination of all epithelioid and sarcomatoid features Differential diagnosis: Synovial sarcoma, other mixed or biphasic tumors

The pathological diagnosis and differential diagnosis of MPM can be very challenging. In a French study, the initial diagnosis of MPM was revised as false positive in 13 % of cases [19]. This can be explained in part by the fact that MPM can present in very heterogeneous forms on the one hand and must be distinguished from benign processes and other tumors, especially metastases of various tumor entities, on the other hand. Such a differential diagnosis can be particularly difficult since mesothelioma-like features can also be found in some lymphomas, thymomas, and carcinomas, etc.

Full-thickness biopsies are required to separate invasive from noninvasive growth patterns and a panel of numerous immunohistochemical markers is required for the differentiation of epithelioid MPM from adenocarcinoma [13].

## Staging

**Fig. 6:**
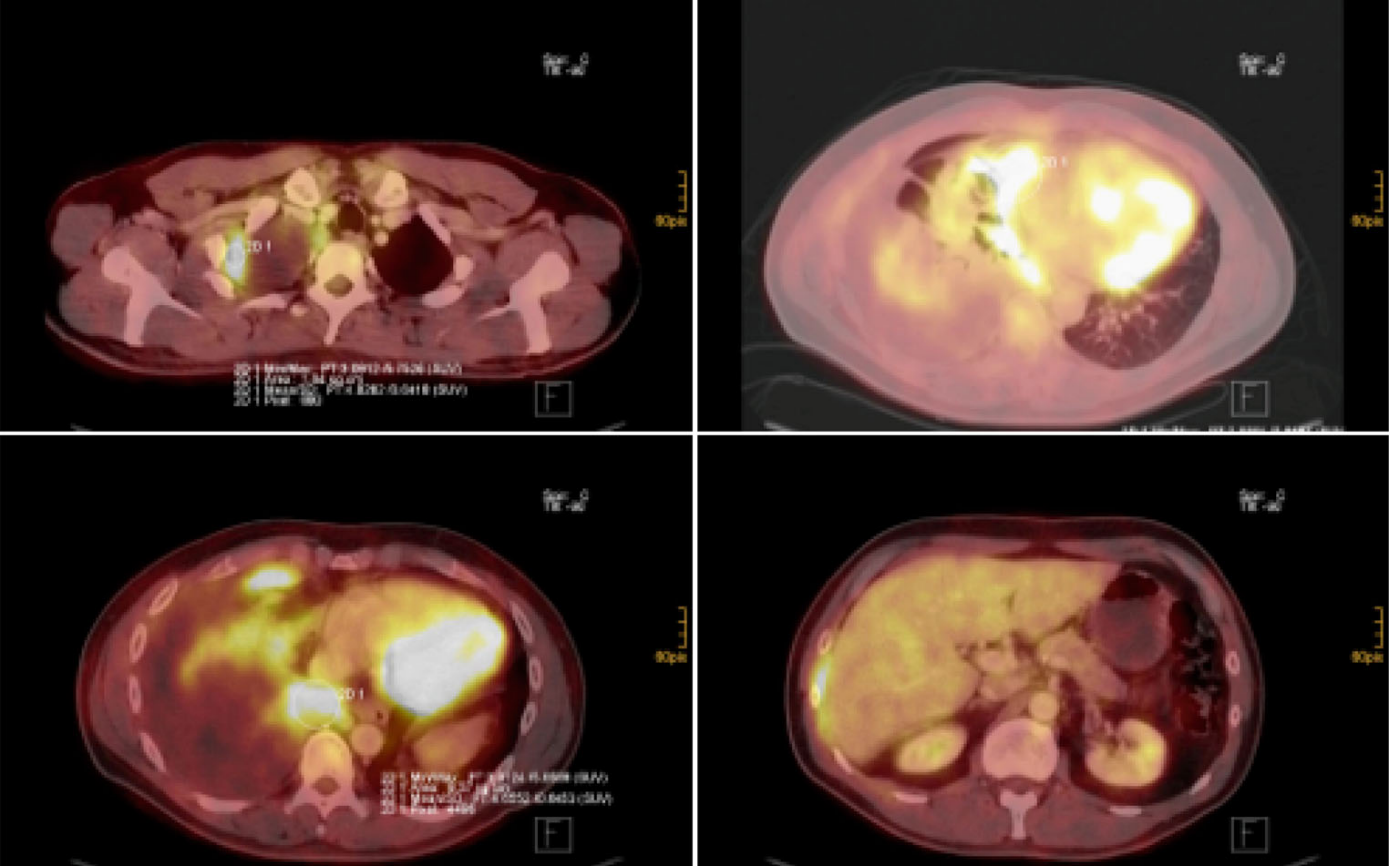
FDG PET-CT images: malignant pleural mesothelioma of the right pleural cavity (various slides of PET/CT fusion imaging). Varoius slides of CT/PET fusion imaging showing pleural tumor apical right (*top left*), involving the visceral and parietal pleura in the pleura costodiaphragmatic area (*bottom left* and *right*) and the pericardium (*top right*)

Both CT and PET-CT (Fig. 6), however, are useful for the further staging.

For all patients, the following assessments for staging and further treatment are required: After initial imaging with CT scan and confirmation of disease via video-assisted thoracic surgery (VATS), a potential candidate for surgical treatment should undergo PET-CT scanning to rule out distant metastasis and involvement of the abdomen and the mediastinal lymph nodes. To rule out the latter, histological confirmation has to be made either by endobronchial/endo-esophageal ultrasonography and transbronchial needle aspiration (EBUS/EUS-TBNA) or mediastinoscopy or VATS according to the lymph node station involvement and the involved side.

If nodes are negative, patients can proceed to induction treatment and should be re-staged with CT or PET-CT. In some cases of unclear involvement of adjacent structures (e. g., chest wall), magnetic resonance imaging (MRI) can be added in order to judge the resectability.

The investigations shown in Table 2 were recommended by many consensus groups.

The most recent available and widely used TNM-based stating system was developed by the International Mesothelioma Interest Group (IMIG) and was also approved by the Union for International Cancer Control (UICC; Tables 3 and 4).

A possible staging algorithm for MPM patients is displayed in Fig. 4.

**Table 2:** Clinical approach and pretherapeutic evaluation according to ERS/ESTS recommendations [13]

**Investigation at presentation (all patients)**	
Demographics	Gender, age, asbestos exposure
Clinical history	Performance status symptoms
Physical examination	Body weight
Radiology	Chest radiograph
Blood tests	–
**Investigations for diagnosis and staging**	
Biopsy of tumor, histological confirmation	– Thoracoscopy – Thoracocentesis – Needle aspiration
Radiology	– CT scan – Brain MRI or CT – Bone scan as required
Pulmonary function tests	–

*CT* computer tomography, *MRI* magnetic resonance imaging

**Table 3:** TNM staging of malignant pleural mesothelioma [24]

T0	No evidence of primary tumor
T1	Tumor limited to the ipsilateral parietal pleura with or without mediastinal pleura and with or without diaphragmatic pleural involvement
T1a	No involvement of the visceral pleura
T1b	Tumor also involving the visceral pleura
T2	Tumor involving each of the ipsilateral pleural surfaces (parietal, mediastinal, diaphragmatic, and visceral pleura) with at least one of the following: – Involvement of the diaphragmatic muscle – Extension of tumor from the visceral pleura into the underlying pulmonary parenchyma
T3	Locally advanced but potentially resectable tumor; tumor involving all of the ipsilateral pleural surfaces (parietal, mediastinal, diaphragmatic, and visceral pleura) with at least one of the following: – Involvement of the endothoracic fascia – Extension into the mediastinal fat – Solitary, completely resectable focus of tumor extending into the soft tissue of the chest wall – Nontransmural involvement of the pericardium
T4	Locally advanced, technically unresectable tumor; tumor involving all of the ipsilateral pleural surfaces (parietal, mediastinal, diaphragmatic, and visceral pleura) with at least one of the following: – Diffuse extension or multifocal masses of tumor in the chest wall, with or without associated rib destruction – Direct diaphragmatic extension of the tumor to the peritoneum – Direct extension of the tumor to the contralateral pleura – Direct extension of the tumor to a mediastinal organ – Direct extension of the tumor into the spine – Tumor extending through to the internal surface of the pericardium with or without a pericardial effusion or tumor involving the myocardium
**Regional lymph nodes (N)**	
NX	Regional lymph node(s) cannot be assessed
N0	No regional lymph node metastases
N1	Metastases in the ipsilateral bronchopulmonary or hilar lymph node
N2	Metastases in the subcarinal or in the ipsilateral mediastinal lymph node, including the ipsilateral internal mammary and peridiaphragmatic nodes
N3	Metastases in the contralateral mediastinal, contralateral internal mammary, ipsilateral, or contralateral supraclavicular lymph nodes
**Distant metastases (M)**	
M0	No distant metastasis
M1	Distant metastasis

**Table 4:** UICC–IMIG staging [24]

UICC staging (7th edition)			
Stage IA	T1a	N0	M0
Stage IB	T1b	N0	M0
Stage II	T2	N0	M0
Stage III	T1, T2 T1, T2 T3	N1 N2 N0, N1, N2	M0 M0 M0
Stage IV	T4 T1–4 T1–4	N0–3 N3 N0–3	M0 M0 M1

### Prognosis

The prognosis for MPM depends on the patient’s age, gender, tumor stage, and geographic region [20]. Other factors such as weight loss and performance status are important for the prognosis as in other tumor entities as well as quality of life and symptom scores. Epithelioid MPM has a better overall prognosis than non-epithelioid histological subtypes. Low hemoglobin levels, high platelet levels, and high serum lactat dehydrogenase (LDH) are prognostically unfavorable characteristics [21]. Numerous new laboratory markers are in evaluation, but no validated data on their prognostic value are available yet [22].
